# Differences in the movement phase condition and sensory inputs on temporal synchronization and continuation during bilateral foot-tapping tasks

**DOI:** 10.3389/fnhum.2025.1518230

**Published:** 2025-01-30

**Authors:** Atsuki Numata, Yasuo Terao, Kenichi Sugawara, Yoshikazu Ugawa, Toshiaki Furubayashi

**Affiliations:** ^1^Physical Therapy Course, Department of Rehabilitation, Faculty of Medical Science and Welfare, Tohoku Bunka Gakuen University, Sendai, Japan; ^2^Department of Medical Physiology, Faculty of Medicine, Kyorin University, Mitaka, Japan; ^3^Graduate Course of Health and Social Services, Kanagawa University of Human Services Graduate School, Yokosuka, Japan; ^4^Department of Human Neurophysiology, Institute of Brain Medical Sciences, Fukushima Medical University, Fukushima, Japan; ^5^Graduate School of Health and Environmental Science, Tohoku Bunka Gakuen University, Sendai, Japan

**Keywords:** time perception, timing, physical therapy, gait rehabilitation, auditory input, tactile feedback, synchronized tapping task, synchronization-continuation task

## Abstract

In the sensorimotor synchronization (synchronized and continuous tapping) task, subjects move their limbs in synchrony with an isochronous tone presented at various tempos and continue tapping at the same pace after the tones have ceased. We investigated the ability of bilateral lower limb motor control for performing this task as a crucial metric for examining motor coordination relevant to human locomotion, such as walking. Here, sensory information such as auditory and tactile inputs is considered to improve the accuracy of sensorimotor synchronization. In this study, we explored the change in tapping variability of rhythmic motor control of the bilateral lower limb with different movement phase conditions in the presence or absence of sensory information. Thirty-three healthy volunteers performed three types of foot-tapping tasks: synchronization-continuation (SC-tap), air-tapping (A-tap), and a combination of both (SCA-tap). Participants were instructed to tap the foot-switch (or perform a similar movement in the A-tap) in synchrony with the tones presented at fixed interstimulus intervals (ISIs) between 500 and 4,800 ms. Taps were performed with either unilateral foot or, in the case of bilateral movements, with both feet, either simultaneously (in-phase) or alternately for bilateral movements (antiphase). The synchronizing tapping error and the inter-tap interval (ITI) were evaluated. The coefficient of variation (CV) of ITI was significantly smaller for the antiphase condition than for the unilateral or in-phase conditions in the SC-tap and SCA-tap tasks. In addition, considering the timing of taps on both sides, the CV was significantly lower for antiphase only in the SC-tap task. The findings indicated that the antiphase condition exhibited superior temporal stability in repetitive lower limb movements. The findings also underscored the significance of tactile feedback from the soles of the feet when stability of rhythmic limb movements unpaced by the tones in antiphase movements was taken into consideration.

## 1 Introduction

Understanding the temporal motor control of the lower limbs holds significant importance in human locomotion, particularly for activities like walking. Gait encompasses not only the ability to maintain a consistent tempo but also to adapt it to different speeds depending on the circumstances. For instance, pedestrians may walk slowly through crowded areas but increase their pace when the traffic signal changes. In social activities, bilateral lower limbs must coordinate to execute seamless alternating motions at varying speeds (e.g., walking, running, or swimming). Thus, assessing interlimb coordination and temporal processing is essential for examining rhythmic lower limb control during gait. Research indicates that when bilateral upper limbs engage in rhythmic movements in opposite directions (antiphase movement), maintaining this pattern becomes increasingly challenging as the pacing frequency rises. Eventually, the movements transition to in-phase movements at higher paces, a phenomenon termed “phase transition.” Phase transition (Haken et al., 1985) is characteristic of voluntary movements of the upper limbs (Scholz and Kelso, 1990; Kelso and Jeka, 1992). Phase transition has also been observed in lower limb movements (Schöner et al., 1990; Kelso and Jeka, 1992), although some studies have not confirmed this (Riek and Carson, 2001). Additionally, research indicates that the transition from walking to running in humans does not adhere to the Haken-Kelso-Bunz (HKB) model (Kao et al., 2003; Seay et al., 2006). This suggests that the motor control mechanism for lower limbs may differ from that of the upper limbs. A semi-automatic control system involving the central pattern generator (CPG) might be involved, especially for the lower limbs, contributing to the greater stability of antiphase movements in the lower limbs compared to the upper limbs.

The bilateral control mechanisms for rhythmic movements of the feet remain unclear, especially regarding how they are influenced by different types of control (voluntary or automatic) and movements involving the upper and lower limbs. To address this, in our previous work (Numata et al., 2022), we utilized a synchronized tapping task, in which participants tapped a button with their fingers or feet in time with tones presented at regular intervals, providing a model for temporal processing (temporal entrainment) (Repp, 2005; Repp and Su, 2013). We investigated differences in temporal processing for bilateral in-phase and antiphase movements using synchronized bilateral finger- and foot-tapping tasks (Numata et al., 2022); normal volunteers tapped with either their fingers or feet in synchrony with tones presented at fixed interstimulus intervals (ISIs; 250–4,800 ms), either simultaneously or alternately. Generally, for trials with short intervals, synchronization errors (SE) were narrowly distributed around 0 ms or were slightly negative (negative asynchrony). Although SE variability increased with longer intervals, the coefficient of variation (CV) of inter-tap intervals (ITI) was significantly lower for antiphase movements compared with unilateral or in-phase movements in foot-tapping but not in finger-tapping. These findings suggest that the preserved temporal synchronization observed in antiphase foot movements, but not finger movements, may stem from distinct neural mechanisms underlying locomotion.

In the context of bilateral lower limb movement, the influence of auditory input and tactile feedback on movement accuracy should also be taken into consideration (Aschersleben and Prinz, 1995). Auditory input aids in generating motor programs for ISIs, while it also serves as feedback for tap timing accuracy (Repp, 2005). Neurotypical individuals can maintain accurate synchronous movement in the range of approximately 200–1,800 ms, exhibiting “negative asynchrony,” in which the tap precedes the sound by a few tens of milliseconds (Fraisse, 1982). Additional tones between the sequences of tones can reduce tap variability (termed “subdivision benefit”) (Repp, 2003). It is also known that following rhythmical or continuous auditory stimuli improves the onset and smoothness of tapping in neurotypical people (McIntosh et al., 1997). From a study using the synchronization-continuation paradigm (Stevens, 1886), which evaluates the accuracy of time interval preservation by tapping synchronously to isochronous tones and continuing to tap at pace after the disappearance of the auditory stimulus, it is known that a shift to self-pace movement and a decrease in mean ITI and variability of ITI occur after the disappearance of an auditory stimulus (Michon, 1967; Semjen et al., 2000). However, patients with Parkinson’s disease (PD) exhibit difficulty in the initiation and continuation of movement based on internal motor programs, whereas they show preserved ability to initiate movement based on external sensory inputs. The former may be caused by dysfunction of the supplementary motor area due to dysfunction of the basal ganglia in PD (Nakamura et al., 1978; DeLong, 1990; Obeso et al., 2000). The latter may be due to sensory input transmitted via the premotor area bypassing the basal ganglia (Praamstra et al., 1998). Previous studies have shown that PD patients may exhibit decreased synchronization ability and accelerated taps in the synchronization-continuation paradigm (Tolleson et al., 2015; Tokushige et al., 2023). While these studies used tasks performed with the upper limb and unilateral movements, it remains unclear which sensory input predominates in rhythmic bilateral lower limb movement, particularly in antiphase movement, and whether stable rhythm control is possible without both sensory inputs (auditory and tactile). Studies using a finger-tapping task demonstrated a decrease in the ability to synchronize with tones and acceleration of taps in the synchronization and synchronization-continuation paradigms in PD (Tolleson et al., 2015; Tokushige et al., 2018). The latter tactile sensory feedback from the finger or the foot is input to the cerebellum and cerebral cortex; it is matched with the predicted timing of the tap and used for the correction of movement (Aschersleben et al., 2001; Aschersleben, 2002). Completely deafferented individuals can tap in phase with a metronome but with a large negative asynchrony (Billon et al., 1996; Aschersleben, 2002). Neurotypical individuals can perform accurately synchronized tapping with negative asynchrony by using both sensory inputs (sound and tactile inputs). However, it remains unclear from the results of the studies mentioned above which sensory inputs have a greater effect on rhythmic movement involving bilateral lower limbs. Specifically, in antiphase movements, is it possible to achieve stable rhythm control without either or both of the sensory inputs?

In this study, we aimed to elucidate the impact of foot movements under varying movement phases and sensory input conditions on rhythmic motor control through synchronized foot-tapping tasks. Specifically, we focused on bilateral motor control of the ankle joint during a synchronized foot-tapping task. We hypothesized that antiphase movements similar to lower limb gait would exhibit greater temporal stability.

## 2 Materials and methods

### 2.1 Participants

We enrolled 33 healthy individuals: five men and six women participated in the synchronization-continuation task (SC-tap task) (mean age ± standard deviation [SD], 29.6 ± 6.0 years); six men and five women participated in the air-tapping task (A-tap task; 29.9 ± 7.2 years); and six men and five women participated in the synchronization-continuation and air-tapping task (SCA-tap task; 33.1 ± 7.9 years) (Table 1). Participants had no history of neurological or orthopedic disease. Foot preference was confirmed using Chapman’s questionnaire (Chapman et al., 1987). All participants were right-foot dominant (laterality index: 12.6 ± 2.0).

**Table 1:** Characteristics of participants.

	Sex		Age (year)	Laterality index	SRT (ms)
Task	Male	Female			
SC-tap task	5	6	29.6 ± 6.0	12.7 ± 2.0	228.2 ± 25.7
A-tap task	6	5	29.9 ± 7.2	12.7 ± 2.4	237.8 ± 29.1
SCA-tap task	6	5	33.1 ± 7.9	12.7 ± 2.0	223.2 ± 19.8
					(mean ± SD)

SRT: simple reaction time.

Written informed consent was obtained from all the participants, and the study was approved by the Ethical Committee of the Tohoku Bunka Gakuen University (No. 16–18).

### 2.2 Experimental procedures

#### 2.2.1 Task procedure

The participants performed a synchronized tapping task in which they had to press a button with their feet in synchrony with the tones presented at regular intervals (ISIs; see also Motor tasks below). Participants sat comfortably on a chair, put their feet on a self-made footrest set at 10° of ankle plantar flexion, and put the ball of their foot on the button.

**Figure 1:**
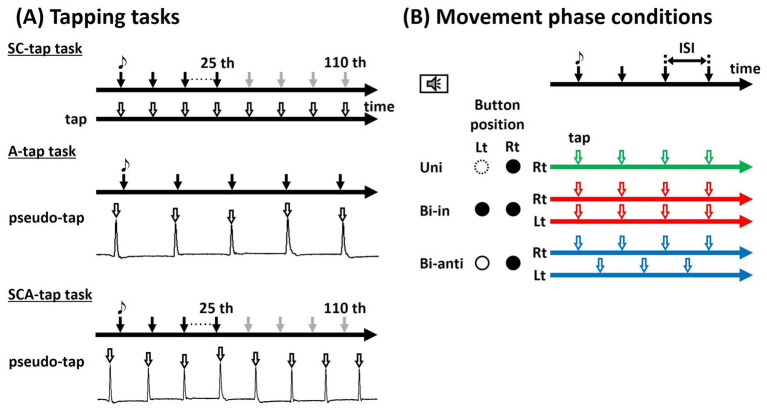
Tapping task. **(A)** Participants were instructed to tap the foot-switch in synchrony with tones presented at fixed interstimulus intervals (ISIs) using either unilateral (Uni; green line) or bilateral feet, under Bi-in (move the bilateral ankles simultaneously) (red line) or Bi-anti (move the bilateral ankles alternately) (blue line) conditions. **(B)** In the SC-tap and the SCA-tap tasks, the sound stimulus disappeared after the 25th tone. In the A-tap task and the SCA-tap task, participants repeatedly moved their feet and pressed the button with the ball of their feet (pseudo-tap).

Participants performed three tasks. Figure 1A shows the schematic chart of the three tapping tasks described below.

To examine the effect of auditory inputs on rhythm generation, we conducted the SC-tap task, in which participants were instructed to press the button using the ball of their foot in synchrony with the tones. In the bilateral movement conditions, each foot pressed a different button. The participants had to synchronize the taps with the tones but not just react to them: the button press had to occur just in time with the tones and not in response to the tone. They were instructed not to move body parts except for the foot during each block. The participants started with the synchronized tapping task with the pacing tones (synchronization period [SP]), but soon after (immediately after the 25th of 110 tones), the tones disappeared. Even after disappearance of the tones, the participants had to continue tapping at the same pace (continuation period [CP]); after that, the participants kept tapping repeatedly while maintaining the tempo learned during the initial 25 taps.

To examine the effect of tactile feedback from the sole by tapping the button on rhythm generation, the synchronized task was also performed without tactile feedback (A-tap task). Participants sat comfortably on a chair, with the lower leg supported by an ottoman below, with only the ankle joint moving freely. Participants were instructed to move their feet in a manner similar to that for pressing the button using the ball of their feet, in synchrony with the sound stimulation (pseudo-tap). To perform movements as similar as possible to the tap in the SC-tap task, tap and pseudo-tap were practiced prior to the measurement. The joint angle of the right ankle was recorded using an electrogoniometer (SG110/A, Biometrics Ltd., Newport, United Kingdom).

In the above two tasks, auditory inputs and tactile feedback from the sole were considered to influence rhythm generation. To study the effect of sensory inputs, a task excluding both sensory inputs (auditory inputs and tactile feedback) was also performed (SCA-tap task). With the same movement settings as for the A-tap task, the tones disappeared immediately after the 25th tone. After that, the participants had to keep tapping repeatedly while maintaining the tempo learned during the initial 25 taps. Similarly to the A-tap task, the joint angle of the right ankle was recorded using an electrogoniometer.

Tasks were carried out in 27 blocks with nine ISIs (500, 600, 900, 1,000, 1,200, 1,800, 2,400, 3,600, and 4,800 ms). Each block consisted of 110 acoustic tones presented with a fixed ISI. The ISI for each block was selected randomly from the nine ISIs using a random number table in a counterbalanced manner. Participants usually managed to match the approximate pace of the tones within the first 10 taps. To avoid muscle fatigue, participants took a brief break (approximately 1–2 min) between the blocks. The simple reaction time (SRT) task was performed after the tapping task (SC-tap, A-tap, or SCA-tap). This was done to measure the time it took for the participants to press the button in response to the tones; that is, as an index of simple somatomotor function.

The acoustic tones (click sounds) were generated by converting electrical signals of an electrical stimulator (SEN-3401, Nihon Kohden, Tokyo, Japan) into acoustic signals using an audio monitor (Model 3,300, A-M Systems, Inc., Sequim, WA, USA). The tone was set to a volume that participants could readily hear (a single-pulse tone with a duration of 1 ms, 50 dB SPL) but that did not induce a startle reaction. Both the time of acoustic sound stimulation and that of the tapping were recorded using a laboratory computer through an A/D converter (PowerLab 8/35, AD Instruments Pty Ltd., Bella Vista, Australia) for off-line analysis (LabChart, AD Instruments Pty Ltd.). We evaluated how precisely the timings recorded by the computer program correlated with those obtained by visual confirmation of the recorded sound waves in the analysis.

In a separate session, participants also performed the SRT task using their dominant foot in an evaluation of their simple sensorimotor function. The SRT task confirmed that the participants’ simple sensorimotor function was within the healthy range (SRT: around 200 ms) and determined whether there were any differences between tasks. They were instructed to press the button as fast as possible after an acoustic “go” signal was presented to them. The ISI of the signals varied randomly between 4,500 and 5,000 ms across trials, as in our previous studies (Tokushige et al., 2018; Numata et al., 2022). We recorded 110 trials in each block.

#### 2.2.2 Movement phase condition

The tasks were conducted under three conditions of movement phases: (1) unilateral movement of the dominant foot (Uni), (2) bilateral synchronous movement of ankles in phase, alternating between bilateral dorsi- and plantarflexion made in the same direction (bilateral in-phase movement: Bi-in), and (3) bilateral synchronous movement of ankles in antiphase alternating unilateral dorsi- and plantarflexion of each ankle but in the opposite direction (bilateral antiphase movement: Bi-anti). Figure 1B is the schematic chart of the movement conditions.

#### 2.2.3 “Off-beat tap” in the Bi-anti condition

For the Bi-anti condition, the dominant foot had to tap in pace with the tones (on-beat tapping), whereas the non-dominant side had to move in between the pacing tones; there was no tone for the non-dominant foot to synchronize with (off-beat tapping). This procedure for Bi-anti tapping may introduce an aspect different from that for the Bi-in tapping; that is, absence of the pacing tone: the dominant foot was paced by a tone but the timing of the non-dominant foot was not paced by the tone. However, we considered that presenting pacing tones for both the dominant and non-dominant limbs would make the task too complicated for the participants, especially at fast tapping rates. This was indeed pointed out in a previous study using a similar bimanual coordination task, in which pacing tones rendered the task complicated for participants to perform at fast paces, making the tapping performance worse than that without pacing tones (Johnson et al., 1998). Although the procedure made the task somewhat similar to the continuation task (for the non-dominant limb, also see Discussion), it is known that healthy participants perform better in synchronization than in continuation tapping; the pattern of impairment does not differ significantly between the continuation and SPs of the task (Pastor et al., 1992; Jones and Jahanshahi, 2011; Wojtecki et al., 2011; Joundi et al., 2012).

In previous studies that compared bilateral in-phase and antiphase movements of the fingers and feet, auditory stimuli were often presented at the timing of each movement on the left and right sides, and the cyclicality of finger flexion and extension movements (plantar flexion and dorsal flexion of the ankle joint) was evaluated (e.g., Haken et al., 1985; Scholz and Kelso, 1990; Kelso and Jeka, 1992; Riek and Carson, 2001). In these experimental settings, “off-beat tapping” does not occur. On the other hand, the purpose of this study was to investigate the generation of time by both lower limbs, so we evaluated the timing of the tap. While the pacing sound is presented at the timings of left and right taps in the Bi-anti condition, pacing tones were presented both during plantar flexion and dorsiflexion in the Uni and Bi-in conditions. When the pacing sounds are given at the same ISI in both conditions, one ISI interval is split into two parts for the Bi-anti condition, whereas the same ISI are not split to halves in the latter conditions. For the above reasons, this study compared the Bi-in and Bi-anti conditions using an experimental setting in which “off-beat tapping” occurs. Therefore, the task in this study differs qualitatively from previous studies that evaluated the periodicity of bilateral movements, and care must be taken when interpreting the results.

### 2.3 Data analysis

Data of the dominant foot were used for all analyses. To compare the timing data of taps among the three movement phase conditions, it was necessary to perform the taps of the dominant side synchronously with the pacing tones. Because there are both on-beat (Uni, Bi-in) and off-beat taps (Bi-anti) for the non-dominant side, only the timing of the taps for the dominant side was analyzed in our study (Numata et al., 2022).

**Figure 2:**
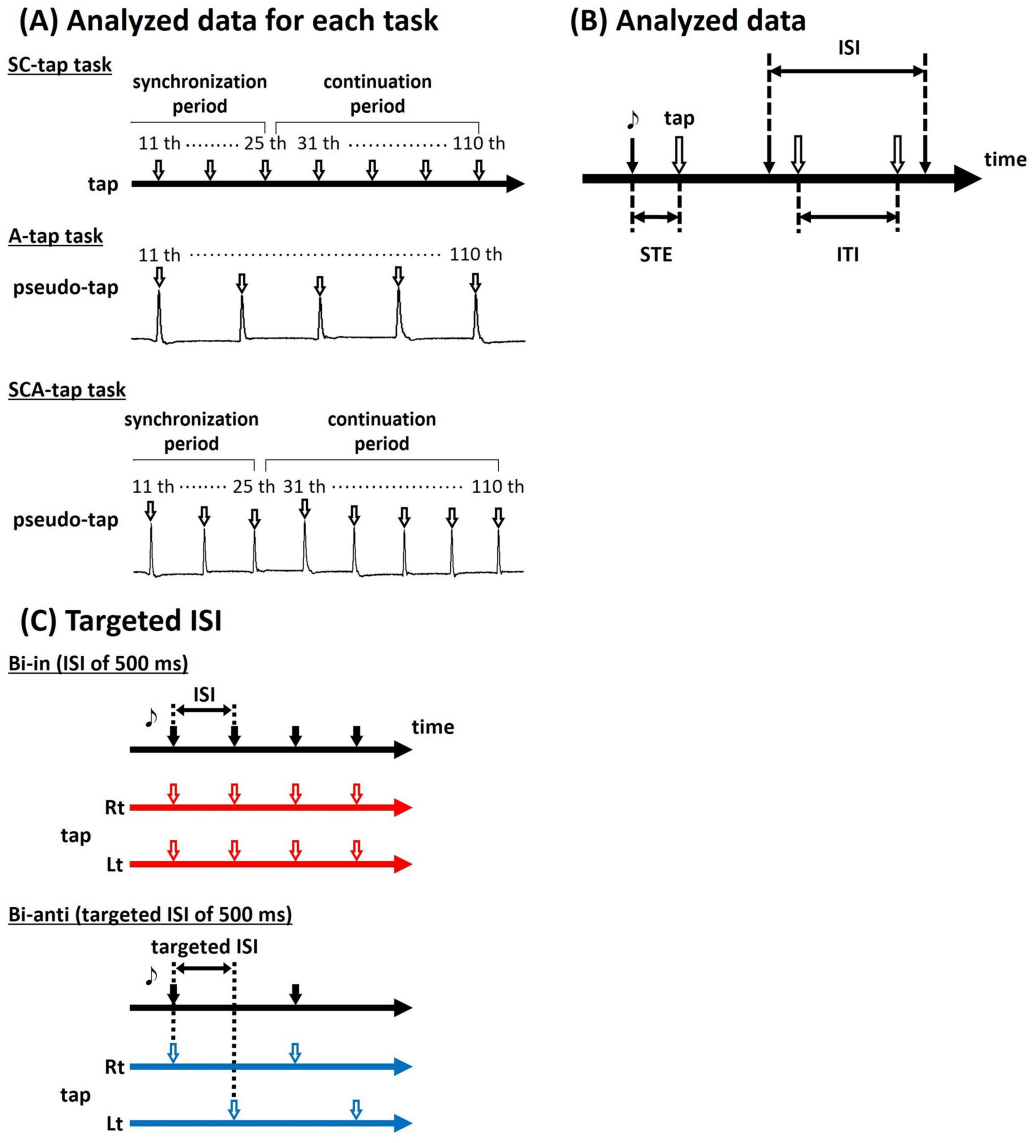
Analyzed data for three tapping tasks. **(A)** For the SC-tap task, we analyzed the recorded data of 95 taps (15 taps of synchronization period and 80 taps of continuation period). For the A-tap task, we analyzed the recorded data of 100 pseudo-taps. For the SCA-tap task, we analyzed the recorded data of 95 taps (pseudo-taps) as with the SC-tap task. **(B)** The timing of the tap and the presented tone (synchronizing tapping error: STE) and the time interval between successive taps (inter-tap interval: ITI) were evaluated. **(C)** In the Bi-anti condition, targeted ISI was defined in order to match the timings of taps on both sides with the other conditions.

Figure 2A shows analyzed data for each task. For the SC-tap task, we analyzed the recorded data of 95 taps (15 taps of SP and 80 taps of CP) for each task after discarding data of 15 taps (initial 10 taps of SP and the initial 5 taps of CP), including those for the timing of the taps. For the A-tap task, we analyzed the recorded data of 100 taps for each task after discarding data of the initial 10 taps, including those for the timing of the pseudo-taps. The timing of pseudo-tap was defined as the timing at which the participant’s joint angle crossed a threshold specified for each participant close to the final range of motion of plantar flexion during repeated ankle movements (plantar-dorsiflexion) of the dominant foot. For the SCA-tap task, we analyzed the recorded data of 95 taps as with the SC-tap task, including those for the timing of the pseudo-taps.

For data analysis, we calculated the ITI; that is, the interval between consecutive taps, in each trial (Figure 2B). In the A-tap task, the time difference between the sound stimulus and the tap (synchronizing tapping error: STE) was also calculated. We also calculated the mean ITI in each task and the CV of ITI, mean STE, and CV of STE in the A-tap task; these values were used for statistical analyses.

For the SRT task, the reaction time was measured from the time of the presented tone to the time of the tap. The mean and SD of SRT were calculated accordingly.

### 2.4 Statistical analyses

The reaction times in the SRT task after each of the three tasks were compared by analysis of variance (ANOVA).

In the SC-tap and SCA-tap tasks, ITI in the CP was divided equally into three time bins (CP1-CP3). Along with the SP as one time bin, the entire duration of the task comprised four time bins in total: SP and CP1 to CP3.

As mentioned above, the three tapping tasks used different measurement methods and data processing. To investigate the effects of tactile feedback, we recorded button tap timing (SC-tap) and measured angles using an electrogoniometer (A-tap and SCA-tap). Because different measurements and data processing were used, the two methods of measuring cannot be directly compared. In addition, because the overall number of trials was specified as 110 based on previous studies, the number of taps used for data processing differed among tasks (95 taps for SC- and SCA-tap; 100 taps for A-tap task), which may have affected the results. Due to these differences, direct comparisons between tasks were not performed, and the analysis in this study was conducted using ANOVA without the task factor.

Data from the SC and SCA-tap tasks (mean ITI and CV of ITI) were analyzed using 3-way repeated-measures ANOVA with the movement phase condition (Uni, Bi-in, Bi-anti), ISI (500, 600, 900, 1,000, 1,200, 1,800, 2,400, 3,600, 4,800 ms), and time bin (SP, CP1, CP2, CP3) as within-subject factors. Data from the A-tap task (mean STE and CV of STE, CV of ITI) were subjected to 2-way repeated-measures ANOVA with the movement phase condition (Uni, Bi-in, Bi-anti) and ISI (500, 600, 900, 1,000, 1,200, 1,800, 2,400, 3,600, 4,800 ms) as within-subject factors. The sphericity of data was assessed by Mauchly’s test; where necessary, the Greenhouse–Geisser correction was used to correct the violation of the assumption of sphericity. *Post hoc* analysis was performed for significant differences detected by the various comparisons using two-tailed *t*-tests with Bonferroni corrections.

In addition, as outlined in the Methods section, this study matched the timings of taps on the dominant side. In the Bi-anti condition, the tap timing on the non-dominant side corresponds to off-beat taps. Therefore, this condition entails twice as many tap timings (i.e., for both the dominant and non-dominant sides) as the other phase conditions. To control for this difference in the number of taps in Bi-anti condition and Uni and Bi-in conditions, we performed a different analysis in which we matched the number of tap timings on both sides between the Bi-anti condition and the Uni and Bi-in conditions (Figure 2C). In this analysis, we defined the “targeted ISI” as the time interval (ISI) between the pacing tones for the dominant and non-dominant limbs, rather than the interval between the tones as before. In the Bi-anti conditions, participants were required to tap in synchrony with the tones presented at the targeted ISI, but for the dominant and non-dominant limbs alternately (see the results section for further details; Numata et al., 2022). Meanwhile, in the Uni and Bi-in conditions, the taps are made by the unilateral limb/bilateral limbs together in synchrony with the pacing tones presented at the same ISI. In this way, the number of tap timings on both sides are made the same. Thus, an ISI of 500 ms corresponds to a 1,000 ms ISI (targeted ISI) in the Bi-anti condition, which is the targeted 500 ms ISI. Similarly, a 2,400 ms ISI in the Uni and Bi-in conditions corresponds to an ISI of 4,800 ms in the Bi-anti condition, with the targeted ISI being 2,400 ms. A range of ISIs, including 500, 600, 900, 1,200, 1,800, and 2,400 ms, was used based on the targeted ISI set by the participants (Numata et al., 2022). We performed 2-way (A-tap task) or 3-way (SC-tap and SCA-tap tasks) repeated-measures ANOVA (factors: movement phase condition: Uni, Bi-in, Bi-anti; ISI: 500, 600, 900, 1,200, 1,800, 2,400 ms; time bin: SP, CP1, CP2, CP3 in the SC-tap and SCA-tap tasks).

Statistical analyses were performed using the commercial software IBM SPSS version 22 for Windows (IBM, Armonk, NY, USA). For all comparisons, *p*-values less than 0.05 were considered statistically significant. However, to reduce the probability of Type I errors, the significance level was adjusted for 2-way and 3-way ANOVAs by regular Bonferroni correction (*α* was divided by the total number of tests (2 main effects and 1 interaction for 2-way ANOVA; 3 main effects and 4 interactions for 3-way ANOVA): 2-way ANOVA: 0.05/3 = 0.017; 3-way ANOVA: 0.05/7 = 0.007) (Cramer et al., 2016). Therefore, for 2-way or 3-way ANOVA, *p*-values <0.017 or 0.007, respectively, were considered statistically significant. Then, the *p*-value was corrected for multiple comparisons, which used the level of *p*-value corresponding to the adjusted level of *p* < 0.05.

## 3 Results

### 3.1 Reaction time in the SRT task

Table 1 shows the SRT. ANOVA revealed that SRTs were not significantly different between groups of participants assigned to the three tapping tasks [SC-tap task: 228.2 ± 25.7 ms; A-tap task: 237.8 ± 29.1 ms; SCA-tap task: 223.2 ± 19.8 ms, *F*(2,29) = 1.025, *p* = 0.372]. This result suggested that the participants’ simple sensorimotor function was within the normal range (SRT: around 200 ms), and that there were no differences between the three tapping tasks.

### 3.2 SC-tap task

**Figure 3:**
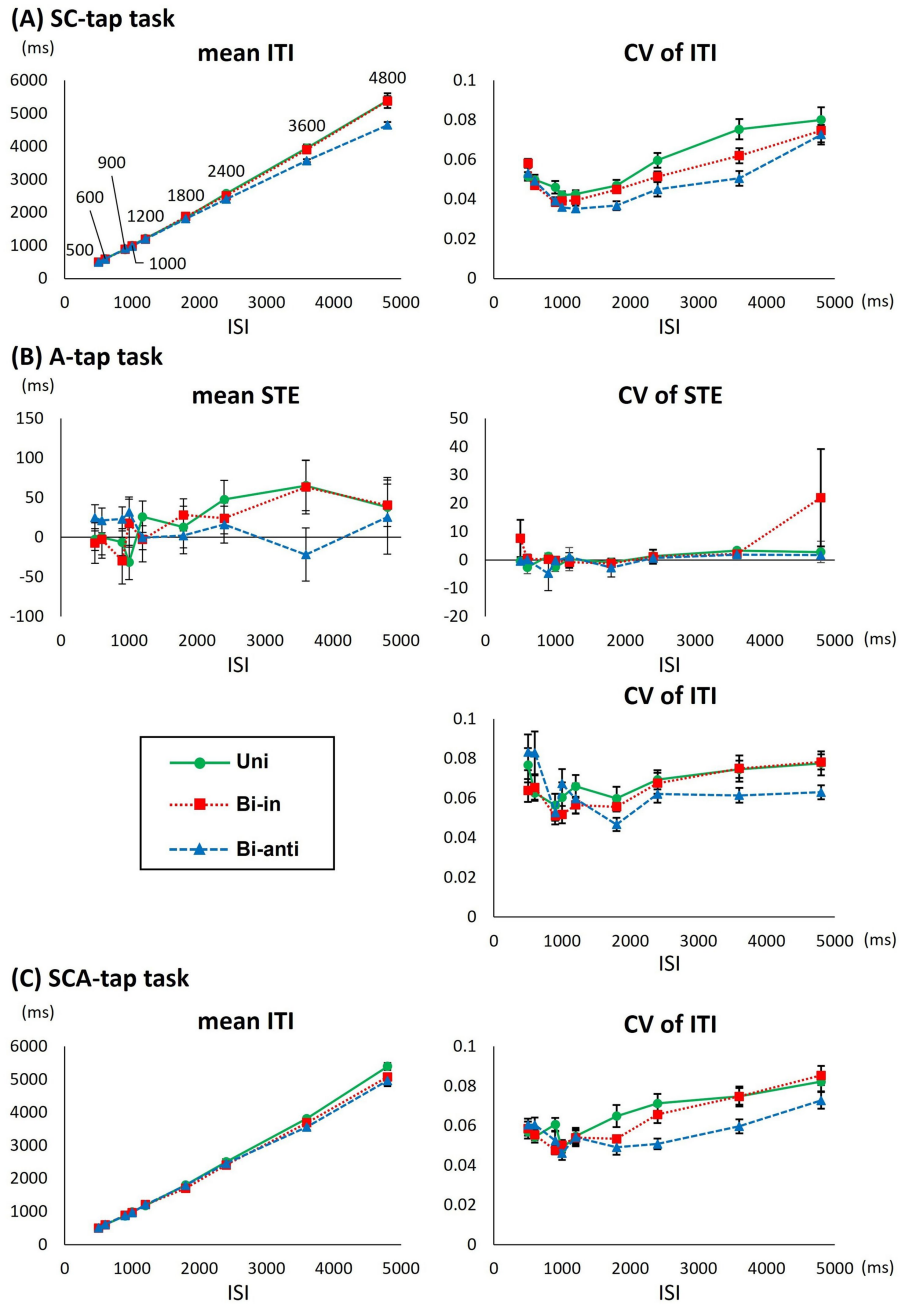
Differences in various parameters for each movement phase condition in the foot-tapping tasks. **(A)** Graph showing the average (mean) inter-tap interval (ITI), the coefficient of variation of the ITI (CV of ITI) of Uni (circles), Bi-in (rectangles), and Bi-anti conditions (triangles) for each interstimulus interval (ISI) in the SC-tap task. Error bars indicate standard error. **(B)** Graph showing the mean synchronizing tapping error (STE), the CV of STE, and the CV of ITI for each ISI in the A-tap task as functions of ISI. **(C)** Graph showing the mean ITI and the CV of ITI for each ISI in the SCA-tap task as functions of ISI.

Supplementary Table 1 shows the mean and CV of ITI, and Supplementary Table 2 summarizes the results of 3-way ANOVAs. Figure 3A compares the tapping performance among the different movement phase conditions at various ISIs in the SC-tap task.

Regarding the mean ITI, 3-way repeated-measures ANOVA showed a significant main effect of ISI (*F*[1.270,12.703] = 493.810, *p* < 0.001) but not of movement phase condition (*F*[1.270,12.698] = 6.544, *p* = 0.019) or time bin (*F*[1.064,10.639] = 3.251, *p* = 0.098). Therefore, movement phase condition did not impact ITI significantly (*p*-values <0.007 were considered statistically significant after corrections for multiple comparisons). There was no significant interaction between the three factors. *Post hoc* analyses at each ISI revealed that the mean ITI increased significantly as the ISI increased (i.e., *p* < 0.001 for all combinations of ISIs).

For the CV of the ITI, a 3-way repeated-measures ANOVA showed a significant main effect for movement phase condition (*F*[2,20] = 9.057, *p* = 0.002) or ISI (*F*[1.478,14.778] = 15.779, *p* < 0.001). There was no interaction among the three factors. *Post hoc* analyses under different movement phase conditions indicated that the CV of the ITI for the Uni condition was significantly larger than that for the Bi-anti condition (Uni: 0.055 ± 0.027, Bi-in: 0.051 ± 0.022, Bi-anti: 0.047 ± 0.022, Uni vs. Bi-in: *p* = 0.185, Uni vs. Bi-anti: *p* = 0.011, Bi-in vs. Bi-anti: *p* = 0.082). Post hoc analyses of the different ISIs revealed that the CV bottomed at 1000 and 1,200 ms and the value of CV increased as the ISI lengthened or shortened (Supplementary Table 3).

### 3.3 A-tap task

Supplementary Table 4 and Figure 3B show the results of the tapping performance under different movement phase conditions in the A-tap task.

Regarding the mean STE, a 2-way repeated-measures ANOVA showed no significant main effect (movement phase: *F*[1.222,12.222] = 0.026, *p* = 0.913; ISI: *F*[8,80] = 1.850, *p* = 0.080) or interaction between the two factors (*F*[4.683,46.833] = 2.430, *p* = 0.052).

For the CV of the STE, a 2-way repeated-measures ANOVA showed no significant main effect (movement phase: *F*[2,20] = 1.627, *p* = 0.221; ISI: *F*[1.759,17.585] = 1.646, *p* = 0.222) or interaction between the two factors (*F*[1.859,18.586] = 0.920, *p* = 0.409).

For the CV of the ITI, a 2-way repeated-measures ANOVA revealed a significant main effect of ISI (*F*[2.993,29.935] = 4.824, *p* = 0.007) but not of movement phase condition (*F*[2,20] = 2.250, *p* = 0.158) or interaction between the two factors (*F*[5.500,55.002] = 2.919, *p* = 0.018). *Post hoc* analyses under different ISIs revealed that the CV at the ISI of 4,800 ms was significantly larger than that at 1,800 ms (*p* = 0.032).

### 3.4 SCA-tap task

Supplementary Table 5 and Figure 3C compare the tapping performance among different movement phase conditions at various ISIs for the SCA-tap task.

Regarding the mean ITI, a 3-way repeated-measures ANOVA showed a significant main effect of ISI (*F*[1.166,11.657] = 514.287, *p* < 0.001) but not of movement phase condition (*F*[2,20] = 3.643, *p* = 0.045) or time bin (*F*[1.036,10.364] = 1.006, *p* = 0.342). There was no significant interaction among the three factors. Therefore, movement phase condition did not impact ITI significantly (*p*-values <0.007 were considered statistically significant). *Post hoc* analyses of each ISI revealed that the mean ITI increased significantly as the ISI became longer (i.e., *p* < 0.001 for all combinations of ISIs, excluding 1,800 ms vs. 2,400 ms: *p* = 0.002).

For the CV of the ITI, a 3-way repeated-measures ANOVA showed significant main effects of movement phase condition (*F*[2,20] = 8.628, *p* = 0.002) and ISI (*F*[3.132,31.321] = 15.467, *p* < 0.001) but not of time bin (*F*[3,30] = 2.865, *p* = 0.053). There was no significant interaction among the three factors. Post hoc analyses under different movement phases indicated that the CV of the ITI for the Bi-anti condition was significantly smaller than that for Uni (Uni: 0.063 ± 0.028, Bi-in: 0.061 ± 0.027 ms, Bi-anti: 0.057 ± 0.026 ms, Uni vs. Bi-in: *p* = 0.200, Uni vs. Bi-anti: *p* = 0.009, Bi-in vs. Bi-anti: *p* = 0.152). Post hoc analyses of the different ISIs revealed that the CV bottomed at 1000 ms and was significantly higher at ISI of 3,600 and 4,800 ms than at other ISIs, whereas there was no significant difference between any other ISIs (Supplementary Table 3).

### 3.5 Changes in the CV of ITI considering the timing of the off-beat tap in the Bi-anti condition (targeted ISI)

**Figure 4:**
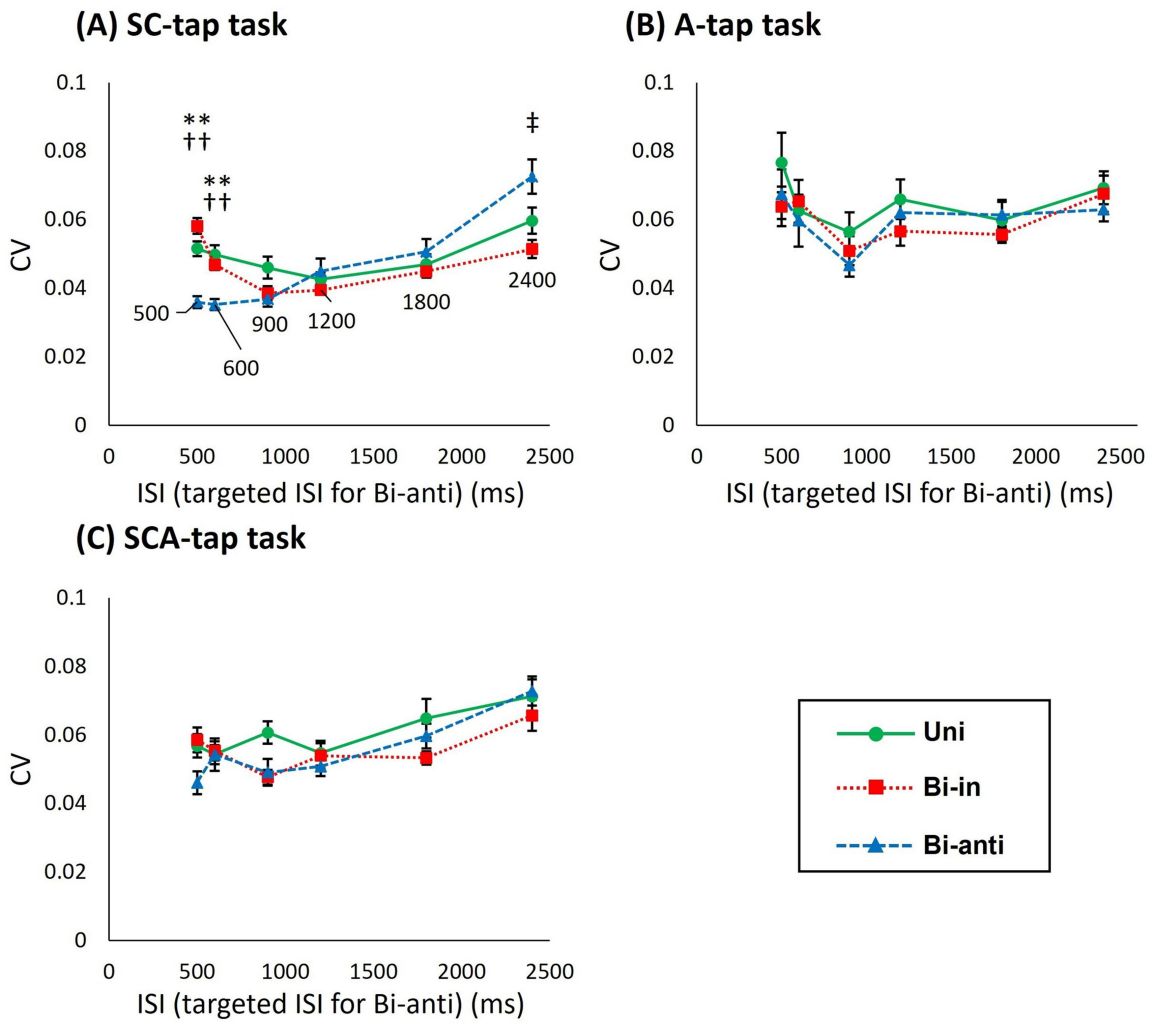
Difference in the CV of ITI for foot-tapping tasks with each movement phase condition matched by targeted ISI on both sides in the Bi-anti condition (dominant/non-dominant). **(A)** Graph showing the coefficient of variation of the inter-tap interval (CV of ITI) of Uni (circles), Bi-in (rectangles), and Bi-anti conditions (triangles) for each targeted interstimulus interval (ISI) in the SC-tap task as functions of ISI. The error bars indicate standard error. **(B)** Graph showing the CV of ITI for each ISI in the A-tap task as functions of ISI. **(C)** Graph showing the CV of ITI for each ISI in the SCA-tap task as functions of ISI. Asterisks indicate significant differences (**, *p* < 0.01 in Uni vs. Bi-anti; ††, *p* < 0.01 Bi-in vs. Bi-anti; ‡, *p* < 0.05 in Bi-anti vs. Bi-in).

Supplementary Table 6 shows the results of the CV of ITI. Figure 4 compares the tapping performances among the different movement phase conditions at ISIs of 500–2,400 ms (targeted ISI for Bi-anti condition) in the three tapping tasks.

In the SC-tap task (Figure 4A), a 3-way repeated-measures ANOVA showed a significant main effect of ISI (*F*[2.023,20.233] = 10.384, *p* = 0.001) but not of movement phase condition (*F*[2,20] = 1.713 *p* = 0.206) or time bin (*F*[3,30] = 0.319, *p* = 0.812). There was significant interaction between movement phase condition and ISI (*F*[10,100] = 7.123, *p* < 0.001). *Post hoc* analyses at the different ISI indicated that the CV of ITI at ISI of 2,400 ms was significantly larger than that at ISIs of 900–1,200 ms (900 ms vs. 2,400 ms: *p* = 0.002, 1,200 ms vs. 2,400 ms: *p* = 0.014). Comparison of CV of the ITI at different ISI showed that CV was significantly smaller in the Bi-anti condition than in the other conditions at ISIs of 500 ms (vs. Uni: *p* = 0.003, vs. Bi-in: *p* < 0.001) and 600 ms (vs. Uni: *p* = 0.001, vs. Bi-in: *p* < 0.001), whereas CV of the ITI was significantly smaller in the Bi-in condition than in the Bi-anti condition at the ISI of 2,400 ms (*p* = 0.012).

For the A-tap task (Figure 4B), a 2-way repeated-measures ANOVA showed no significant main effect (movement phase: *F*[2,20] = 2.617, *p* = 0.098; ISI: *F*[2.404,24.036] = 4.097, *p* = 0.024) or interaction between the two factors (*F*[10,100] = 0.675, *p* = 0.745).

For the SCA-tap task (Figure 4C), a 3-way repeated-measures ANOVA showed a significant main effect of ISI (*F*[5,50] = 7.971, *p* < 0.001) but not of movement phase condition (*F*[1.214,12.142] = 1.714, *p* = 0.218) or time bin (*F*[1.838,18.832] = 3.566, *p* = 0.052). There was no significant interaction among the three factors. Post hoc analyses of CV of the ITI at different ISIs indicated that the CV of the ITI at ISI of 2,400 ms was significantly larger than that at 500–900 ms and 1,800 ms (compared to 2,400 ms, 500 ms: *p* = 0.043, 600 ms: *p* = 0.005, 900 ms: *p* = 0.018, 1,800 ms: *p* = 0.007).

## 4 Discussion

The present study demonstrated that the performance of three foot-tapping tasks varied according to ISIs and movement phase conditions (in-phase and antiphase), with the Bi-anti condition being more stable than other conditions, regardless of the presence or absence of sensory inputs (auditory and tactile). Specifically, the variability of ITI was significantly lower in the Bi-anti condition than in other movement phase conditions. Additionally, in the targeted ISI analysis within the Bi-anti condition, tap variability was notably reduced compared with other phase conditions for shorter ISIs (500–600 ms). These findings support the hypothesis that motor control for temporal synchronization and continuation differs between antiphase movement and other phase conditions in lower limb tasks.

As described in the Methods section, due to the qualitative and methodological differences among the three tapping tasks, the analysis was conducted without direct comparison among tasks and without including task factors. Therefore, care should be taken in interpreting the results, and the differences in results between tasks were speculated. For example, for the SC-tap task, we analyzed the recorded data of 95 taps (15 taps of SP and 80 taps of CP) for each task after discarding data of the 15 taps (initial 10 taps of SP and the initial 5 taps of CP), including those for the timing of the taps. The A-tap task is provided with fewer tones for synchronization or initial entrainment, although it could potentially narrow the distribution of asynchrony (i.e., 0 ms). In addition, previous research has shown that repetitive movement tasks such as circle drawing, which do not involve tactile input, are carried out based on control of movement parameters such as tangential velocity and muscle strength, rather than being controlled by the internal clock mechanism of central nervous system, and that they show different results from tapping tasks (they do not follow the Wing and Kristofferson (1973) model, do not show negative lag-one covariance, do not correlate with the timed interval variability seen in finger-tapping, and are not impaired even in patients with cerebellar disorders that interfere with the tapping task (Robertson et al., 1999; Zelaznik et al., 2000; Zelaznik et al., 2002; Spencer et al., 2003; Zelaznik et al., 2005; Studenka and Zelaznik, 2008; Repp and Steinman, 2010; Studenka et al., 2018), and are described as “non-clock timing”). Thus, there was a qualitative difference between the SC-tap task and the A-tap and SCA-tap tasks. However, in the A-tap and SCA-tap tasks in this study, participants were asked to learn the tap movement and then pseudo-tap was measured. Studenka et al. (2018) reported that both taps and pseudo-taps follow the Wing and Kristofferson model, and we consider that the pseudo-taps in this study also represent a motor task similar to the tap movement. Additionally, it is known from previous studies that the number of reactive taps (close to SRT, around 200 ms) increases with longer ISIs in synchronized tapping (Mates et al., 1994; Matsuda et al., 2015; Tokushige et al., 2018). We reported on this in our previous study (Numata et al., 2022), in which we found generally similar trends in the upper and lower limbs. In the A-tap task of the present study, we observed the same results as in the previous studies, with longer ISIs resulting in more STE and greater rhythmic variability. This also suggested that the A-tap task could be performed as a motor task, similar to the tapping task. The fact that we observed different effects on rhythm stability for each movement phase condition across the three tasks with different auditory and tactile input conditions suggests that sensory inputs affect rhythm generation in the lower limbs. The differences between the tasks were described, focusing on the results of each task.

### 4.1 Specificity of Bi-anti condition for rhythm encoding and retention

In the SC-tap and SCA-tap tasks, the CV of ITI was significantly lower in the Bi-anti condition than in the other movement phase conditions, including the Uni condition. Especially in the SC-tap task, the CV of targeted ISI was smaller in the Bi-anti condition than in the other conditions at ISIs of 500–600 ms. In contrast, there was no difference between the movement phase conditions in the A-tap task, in which participants performed tap-like movements (pseudo-tap) in the air with their feet, not contacting the foot-switch. Consistent with Numata et al. (2022), the findings suggest that stable, repetitive, alternating movements of bilateral lower limbs can be made accurately, even when there is no auditory information. In contrast, the lack of difference between the movement phase conditions in the A-tap task suggests that tactile feedback from the plantar surface is important for maintaining stability of Bi-anti. Although it is considered that the internal clock plays a more important role than tactile feedback for adjusting the ITI according to the prediction based on ISIs (Aschersleben and Bertelson, 2003; Repp and Penel, 2004), the results of the present study suggest that tactile feedback from the plantar surface is important for rhythm generation for alternating bilateral lower extremity movements. Thus, the results of this study point to the importance of tactile feedback from the plantar surface of the foot for generating and maintaining stable rhythms of bilateral lower limb alternating movements. However, it is undeniable that there were tests with insufficient power for the ANOVA of the A-tap task (Supplementary Table 2), and the regular Bonferroni correction used to reduce the risk of Type I error in 2-way and 3-way ANOVA may have made the test results conservative. On the other hand, significant main effects and interactions were observed for the SC- and SCA-tap tasks. In addition, Numata et al. (2022) conducted an experiment with a design similar to the A-tap task in this study and obtained results similar to those of the SC-tap task in this study. The main differences between the foot-tapping task in Numata et al. (2022) and the A-tap task in the present study are the presence or absence of tactile feedback and the difference in the recording method used (button or goniometer). Based on the above, it is considered that the A-tap task did not show significant differences among the movement phase condition due to the absence of tactile feedback, although the low power of the test cannot be denied.

For SC-tap task, tactile feedback from the plantar surface during taps may contribute to the stability of the Bi-anti condition; for the alternating movement of the lower limbs, tactile feedback from the plantar surface of the foot may be utilized by the unique control mechanism (e.g., CPG) to generate stable, patterned, rhythmic movements of the lower limb. Indeed, changes in peripheral sensory input, such as the sensation of load, Group Ia afferent input from hip flexor stretch during the late stance phase, and decrease in Group Ib afferent input from the triceps muscle associated with early leg release (Rossignol et al., 2006; Grey et al., 2007; Zwergal et al., 2012), are considered important for the generation of walking rhythm by CPG. However, the interaction between voluntary descending control by the motor area, including the premotor area, and ascending input from the periphery are considered important for driving CPG in human walking (Takakusaki, 2013). It is unclear whether CPG is also driven in the unloaded movement task of the ankle alone in this study, and further neurophysiological research is needed (also see Limitations and Conclusion). However, considering the specific neural activity observed in antiphase movements (Zehr et al., 2009; Hiraoka et al., 2014), it is possible that descending projections from the supraspinal central nervous system modulated the peripheral neural activity coming from reciprocal phasic movements, thereby generating temporally stable rhythmic movements. Tactile feedback from the plantar sole (especially of the forefoot) may be a more important source of information than muscular sensation for the stable temporal regulation of rhythmic movements of the lower limb involved in walking (Rossignol et al., 2006).

Spencer et al. (2003) conducted a study comparing the timing of production between discontinuous and continuous repetitive movements. They utilized repetitive tapping tasks, with participants tapping in the “air” for the continuous task and tapping on the table for the discontinuous task. They proposed that the neural structures responsible for the temporal characteristics of continuous movements, arising from repetitive movement (continuous task), are distinct from those governing discontinuous movements, which involve an explicit representation of the temporal goal, a function attributed to the cerebellum. The same contrast may apply to the tapping task performed in the air and that performed using a button press, as in the present study. Future studies using the same tasks may be warranted in patients with the cerebellar disorders.

### 4.2 Effect of off-beat tap on the Bi-anti condition (targeted ISI)

The tactile feedback produced by off-beat tapping on the non-dominant side in the Bi-anti condition likely contributed to the stability of the ITIs, offering additional information for estimating the ISIs. In the finger-tapping task, addition of sound stimuli, which subdivide the ISIs, has been shown to enhance the accuracy of rhythm generation when presented along with the tone stimuli at the ISIs (Repp, 2003; Semjen et al., 1992). However, performing an off-beat tap necessitates temporal processing and the execution of a motor program to generate a tap at half of the ISI duration in the absence of a sound stimulus. This situation is similar to the CP, in which taps are executed after the tone has ceased, i.e., in the absence of sound stimuli and relying on the memory of ISIs learned up to that point. Therefore, performing an off-beat tap precisely at the midpoint of the intended ITI can be more challenging than an isochronous tap to a paced tone.

To determine whether ISI segmentation by off-beat tap enhances or inhibits generation and retention of tapping rhythm, we performed 2-way or 3-way repeated-measures ANOVA, matching the timing of both tap sides across phase conditions (see Methods section). As mentioned above, the number of taps in the Bi-anti condition was twice that in the other tap conditions because the taps on the dominant side (right side) are made in synchrony with the tones in the tasks of the present study, while the off-beat tap is made by limbs on the other side (left side). We defined the time interval between the left and right taps (i.e., half of ISI duration) in the Bi-anti condition as *targeted ISI*. For the same targeted ISI, the number of taps per cycle is the same in the Bi-anti condition as in the Uni and Bi-in conditions, whereas the number of sound stimuli per one ISI duration is half that in the Bi-anti condition. If the CV of ITI in Bi-anti condition (targeted ISI) is smaller than that in Uni and Bi-in conditions, Bi-anti is considered to be stable regardless of ISI segmentation; that is, it is stable even with fewer sound stimuli. The results of the analysis showed that the Bi-anti condition was significantly more stable than the other phase conditions at the shorter ISIs of 500–600 ms in the SC-tap task. In addition, the Bi-in condition was significantly more stable than the Bi-anti condition at ISI of 2,400 ms. In contrast, there was no difference between phase conditions in the A-tap and SCA-tap tasks. Interestingly, the CV of the Bi-anti condition showed significant temporal stability despite the ISI being twice that of the other conditions at the short ISIs (500–600 ms) of the SC-tap task. This result is similar to the results of a previous synchronized tapping task (Numata et al., 2022) and suggests that alternating movements of the lower limb were stable, even at fast movement frequencies (Riek and Carson, 2001). In contrast, the Bi-anti condition was less stable than Bi-in at ISI of 2,400 ms. This is because the ISI of 4,800 ms corresponded to the targeted ISI of 2,400 ms in the Bi-anti condition, which was well above 3 s. The 3-s rule of Pöppel states that the duration of the “subjective present” or “experienced moment” is about 3 s (Pöppel, 1997). This implies that the upper limit of time interval between two tones should be shorter than about 3 s for them to be considered continuous, which is also the longest interval that the subject can generate a rhythm in pace with; above this limit, it is considered difficult to generate rhythm in pace with the tones.

### 4.3 Lack of “bimanual advantage” in lower limbs movement

Across the three tasks conducted in the present study, there were no notable differences between the Uni and Bi-in conditions, except at the ISI of 900 ms, as indicated by the analysis regarding the targeted ISI in the Bi-anti condition. This result suggested that the increase of tactile feedback from both sides did not significantly influence the generation and retention of rhythm in lower limb movements compared to that from one side only.

“Bimanual advantage” has been recognized in tasks involving finger-tapping (Franz et al., 1996; Helmuth and Ivry, 1996; Ivry and Hazeltine, 1999; Drewing et al., 2002), a phenomenon in which tapping is more temporally stable with bilateral index fingers than unilateral tapping, which is attributed to differences in the amount of sensory input. Studenka et al. (2018) conducted a tapping experiment with a synchronization-continuation paradigm using bilateral fingers at an ISI of 800 ms. The findings indicated bimanual advantage, with the variability of ITI notably larger in the air-tap condition than in the actual tapping condition. In the present study, we also found a significant difference between Uni and Bi-in conditions in the SC-tap task with an ISI of 900 ms, partially supporting the results of Studenka et al. (2018). However, these studies used ISIs of 400–800 ms, whereas the present study found significant differences between the Uni and Bi-in conditions only at the ISI of 900 ms in the SC-tap task. This suggested that the bimanual advantage for rhythmic motor control of the lower limb may be less pronounced at higher movement frequencies than that of the upper limbs (i.e., ISIs of 500–600 ms).

The limited effect of “bimanual advantage” on the lower limbs may be related to differences in tactile sensitivity. The density of receptors related to tactile sensation, the resolution of afferent fibers, and the size of the area in the sensory cortex are all greatest in the fingers (Johansson and Vallbo, 1983; Johnson and Phillips, 1981; Nelson et al., 1980; Pons et al., 1985). Therefore, the finger-tapping task was considered more susceptible to influence by tactile feedback than the foot task.

The distinction between the upper and lower limbs may reside in their primary roles in daily activities; the upper limbs are typically engaged in tasks requiring precise movements irrespective of phase, whereas the lower limbs emphasize stability during antiphase movements. The joints of the lower limbs primarily have a load-bearing function. In various daily life scenarios, bilateral limbs tend to move in opposite directions rather than in the same direction, whereas instances of bilateral repetitive flexion and extension in the same direction are relatively rare (as in situations such as squatting and rowing, certain weightlifting exercises). The findings of the current study may align with the characteristics of lower limb movements, which necessitate bilateral limbs to execute temporally stable rhythmic movements in an antiphasic rhythm (Kao et al., 2003; Seay et al., 2006).

### 4.4 Changes in ITI during the CP of SC-tap and SCA-tap tasks

The results of this study showed no main effects or interactions related to the time bin factor. In other words, there was no clear shortening or lengthening of ITI in the CP relative to that in the SP among the three movement phase conditions. Although some participants showed a tendency to gradually shift away from and toward the pacing rhythm during the CP, this could not be considered as retention or adjustment, and no common tendency was observed among the participants. Rather, each participant may have shifted to his/her own pace or preferred rate.

In previous studies, self-paced movements were more likely to converge to a certain fixed period (250 or 500–600 ms) (Collyer et al., 1992, 1994; Collyer et al., 1997; Drake et al., 2000) and were even more likely to be perceived and acted upon at integer multiples of that period (Nagasaki, 1987; Collyer et al., 1992, 1994; Collyer et al., 1997; Drake et al., 2000). Short durations are likely to be overestimated and long durations are likely to be underestimated, with the boundary generally considered to be around 500 ms. It has also been reported that CV gradually decreases (stabilizes) with repeated trials (Helmuth and Ivry, 1996). However, in the present study, no clear and consistent trend as described above was observed. This may be ascribed to an individual difference or may be considered characteristic specific for the lower limb movements, which would merit further verification in future studies.

### 4.5 Limitations and conclusion

One limitation of this study is that sensory input from the periphery was not entirely blocked. Previous studies using finger-tapping tasks after peripheral nerve block demonstrated reduced tapping accuracy and increased variability (Aschersleben et al., 2001). Hence, it is necessary to investigate whether stable rhythm generation can be achieved even when sensory inputs from the periphery are totally blocked by nerve compression or anesthesia. This will clarify whether the temporal stability of repetitive movements persists in the absence of proprioceptive feedback. Second, the results obtained in this study do not include neurophysiological data. The high temporal stability of tapping in the Bi-anti condition is discussed in terms of the involvement of neural mechanisms specific to alternating movements of the lower limb. However, validation using neurophysiological measures such as the H-reflex and transcranial magnetic stimulation methods is needed to clarify this point. Third, there are limitations in the statistical analysis. The three tapping tasks in this study recruited different but relatively few participants. Considering the functions of the basal ganglia and cerebellum involved in time perception and the 3-s rule (Pöppel, 1997), a wide range of ISIs from milliseconds to just under 5 s were selected. Even considering the off-beat tap in the Bi-anti condition, it was necessary to perform twice as many ISIs as in the Uni and Bi-in condition. As a result, there was a test that seemed to lack the power of ANOVA (Supplementary Table 2). In addition, the regular Bonferroni correction used to reduce the risk of Type I error in 2-way and 3-way ANOVA have made the test results more conservative, i.e., the risk of Type II error has increased. Moreover, because different data analyses (tapping or pseudo-tapping) were conducted for each task, task factors were not included in the ANOVA. Therefore, only the characteristics of each tapping task are discussed. To directly compare tasks, it is necessary to include the task factor in the analysis by adjusting all tasks to the same settings, such as increasing the number of subjects or reducing the number of task and ISI levels. However, the SC- and SCA-tap showed significant main effect and interaction after adjustment for the regular Bonferroni correction. Furthermore, in our previous study (Numata et al., 2022), we conducted an experiment with a design generally similar to the A-tap task and obtained results similar to the SC-tap task in this study. The main differences between the foot-tapping task of Numata et al. (2022) and the A-tap task of the present study were the presence or absence of tactile feedback and the method of data recording (button switch or goniometer). This suggests that the absence of tactile feedback in the A-tap task resulted in rhythm generation relying solely on the internal clock mechanism, and that no differences were observed between the movement phase conditions. Finally, care should be taken in interpreting the results of the “targeted ISI” analysis. In order to clarify the effect of “off-beat tap” in the Bi-anti condition, ISI combinations were adjusted between the movement phase conditions. This adjustment allowed us to compare the CV of ITI, considering the ITI between the both sides. It should be noted, however, that the Uni and Bi-in conditions are not strictly comparable to the Bi-anti condition, as the time required for each block and the total number of taps performed did not change.

The aim of the present study was to explore how bilateral movement phase and the presence or absence of sensory information influence rhythmic motor control of the lower limb. The results indicated that tapping variability was smaller in the Bi-anti condition for both the SC-tap and SCA-tap tasks compared with other phase conditions. Furthermore, the targeted ISI analysis, which matched the timing of bilateral taps, revealed significantly greater stability in the Bi-anti condition, particularly for shorter targeted ISIs in the SC-tap task. These findings underscore the superior stability of the Bi-anti condition in repetitive lower limb movements and also point to the importance of tactile feedback from the sole for movements such as those required for walking. The bilateral foot-tapping task employed in this study can assess the temporal processing ability specific to lower limb rhythm generation, crucial for walking. Future research is warranted to investigate how the performance of this task is affected in patients with PD and spinocerebellar ataxia, conditions associated with impaired temporal processing, and to explore its potential application in gait rehabilitation.

## Data Availability

The raw data supporting the conclusions of this article will be made available by the authors, without undue reservation.
